# *Rabdosia rubescens* Inhibits CYP7A1 Expression and Induces Autophagy to Reduce Alcohol‐Induced Liver Damage in Mice

**DOI:** 10.1155/cjgh/9507266

**Published:** 2025-10-10

**Authors:** Xi Zou, Wei Chen, Yidan Shao, Tingting Shi, Yunling Ke

**Affiliations:** ^1^ Department of Pharmaceutical Preparation, The Hangzhou Xixi Hospital Affiliated to Zhejiang Chinese Medical University, Hangzhou, 310023, Zhejiang, China; ^2^ The Fourth School of Clinical Medicine, Zhejiang Chinese Medical University, Hangzhou, 310023, Zhejiang, China, zcmu.edu.cn

**Keywords:** alcoholic liver injury, autophagy, cholestasis, CYP7A1, *Rabdosia rubescens*

## Abstract

**Objective:**

Investigating the antialcoholic liver injury properties of *Rabdosia rubescens* and its effect on the expression of CYP7A1 and levels of autophagy.

**Materials and Methods:**

Male C57BL/6 mice were randomly divided into three groups, namely, the control group (Lieber–DeCarli standard diet), model group (Lieber–DeCarli ethanol diet), and treatment group (*Rabdosia rubescens* at concentrations of 50 mg/kg, 100 mg/kg, and 200 mg/kg). The mice were fed their respective diets for 10 days, with the treatment group receiving the corresponding concentration of *Rabdosia rubescens*. On the 11th day, all the mice except those in the control group were given 30% EtOH. Nine hours later, the mice were sacrificed and their blood serum and liver tissue were collected for analysis. The effect of *Rabdosia rubescens* on alcohol‐induced liver damage was evaluated by testing serum biochemical indicators, liver bile acid content, liver tissue histopathological changes, liver LC3 and p62 immunohistochemical staining, liver inflammatory factors, and CYP7A1, SREBP, FAS, LC3, Beclin‐1, ATG7, and p62 expressions.

**Results:**

Compared with the model group, *Rabdosia rubescens* was found to significantly reduce the levels of ALT, AST, TG, TC, and TBA in the serum of mice with alcoholic liver injury (*p* < 0.05 or *p* < 0.01). *Rabdosia rubescens* was found to significantly reduce the level of apoptosis in H&E‐stained liver tissue sections. *Rabdosia rubescens* also significantly reduced the levels of TNF‐α and IL‐1β in the livers of mice (*p* < 0.001). *Rabdosia rubescens* was found to induce the expression of LC3, Beclin‐1, and ATG7 proteins and mRNA, while inhibiting the expression of CYP7A1, SREBP, FAS, and p62 proteins and mRNA (*p* < 0.05 or *p* < 0.01).

**Conclusion:**

*Rabdosia rubescens* has been shown to relieve alcohol‐induced liver damage in mice by reducing levels of CYP7A1, alleviating cholestasis and reducing inflammation. It can also reduce alcohol‐induced liver damage by decreasing fat synthesis and inducing autophagy.

## 1. Introduction

Alcoholic beverages are prevalent worldwide, and according to the latest World Health Organization Global Status Report on Drinking and Health, more than two‐fifths of people over the age of 15 years, approximately 2.3 billion people, consume alcohol. The average amount of alcohol consumed is 33 g of pure ethanol per person per day​ [1]. Additionally, 90% of people who consume more than 16 g of alcohol a day will develop liver disease. Long‐term alcohol consumption can lead to alcohol‐related liver disease (ALD) [2, 3]. Alcohol has become a leading cause of chronic liver disease, with its incidence and mortality rates continuing to rise. According to statistics, about 2,460,000 people die of chronic liver disease every year, with alcohol consumption responsible for about 50% of these deaths [4].

Fat accumulation is a key feature of ALD. This condition is characterized by the accumulation of lipid droplets in hepatocytes, which mainly comprise triglycerides (TG) and cholesterol (CHO) [5]. The causes of liver fat accumulation associated with ALD include an increased fat supply, enhanced de novo lipogenesis, reduced fat clearance, and decreased fatty acid β‐oxidation. De novo lipogenesis is the main source of liver fat among these [6]. The transcription factor sterol regulatory element‐binding protein 1c (SREBP‐1c) controls the expression of genes involved in the production of fat in the liver [7]. Acetaldehyde can directly increase SREBP‐1c expression, thereby promoting fat accumulation in the liver [8]. In healthy livers, farnesoid X receptor (FXR) typically suppresses SREBP‐1c; however, in patients with ALD, the inhibition of FXR results in ongoing fat production [9]. Damage to the SREBP‐1c gene can reduce fat accumulation in the liver and slow the progression of ALD [10]. The presence of excess fat in the liver causes it to react with the active oxygen produced by the metabolism of alcohol, resulting in the formation of toxic aldehydes. These aldehydes trigger liver cell apoptosis and facilitate liver fibrosis, thus accelerating the progression of chronic liver disease to cirrhosis [11]. Targeting SREBP‐1c to suppress the synthesis of hepatic cell fat is a promising therapeutic approach for ALD.

Hepatocytes can selectively clear damaged mitochondria and excess lipid droplets from cells through autophagy, maintain the stability of the internal environment of hepatocytes, enable adaptation to metabolic changes in the internal environment induced by alcohol, and promote cell survival [12]. The level of autophagy in the liver significantly increased in mice exposed to acute alcohol. This is a form of self‐protection for liver cells under stressful conditions [13]. This augmentation in autophagy can eliminate damaged mitochondria and diminish liver cell apoptosis. However, long‐term alcohol consumption damages liver cell autophagy, resulting in significantly fewer autophagy substrates being hydrolyzed [14]. The reduction in autophagy levels caused by this process indirectly promotes the accumulation of fat in liver cells. Targeting the induction of autophagy presents a promising method for treating ALD. While pharmacological inhibition of autophagy significantly increases hepatocyte apoptosis, activation of autophagy can alleviate alcohol‐induced liver damage [15].

As the accumulation of bile acids in the liver is toxic, the body strictly controls their production [16]. The FXR functions as a negative regulator of bile acid synthesis. An elevation in bile acid levels within hepatocytes triggers FXR, which in turn decreases the bile acid concentration in hepatocytes by encouraging transport and inhibiting synthesis [17]. CYP7A1 is the rate‐limiting enzyme in the synthesis of bile acids. FXR negatively regulates this process by suppressing the expression of CYP7A1 [18]. When it comes into contact with excessive amounts of bile acids, FXR in the gut promotes the secretion of FGF15. FGF15 then acts on hepatocytes, inhibiting the expression of CYP7A1 [17]. The alternative synthetic pathway for bile acids is crucial in newborns, whereas the classic pathway generates 80% of bile acids in adults [16]. The reduced expression of the CYP7A1 enzyme significantly restricts the rate at which bile acids are synthesized in hepatocytes. Cholestasis occurs at each stage of ALD, accompanied by changes in the content and composition of bile acids [19]. Inactivation of FXR in the livers of ALD patients leads to a significant increase in CYP7A1 expression [20]. Furthermore, FXR promotes fat production, providing sufficient raw materials for bile acid synthesis. Together, these two factors promote cholestasis in ALD patients. CYP7A1 is also a key target for ALD treatment, and studies suggest that inhibiting CYP7A1 expression can alleviate cholestasis in the liver [21].

Traditional Chinese medicine has significant advantages in treating chronic liver disease due to its complex components and numerous therapeutic targets. *Rabdosia rubescens* is a commonly used over‐the‐counter Chinese medicine with good anti‐inflammatory, antibacterial, antitumor, antiangiogenic, and antiasthmatic effects [22]. *Rabdosia rubescens* has been utilized in traditional Chinese medicine for over a century. It is available in various forms, such as tablets, drops, syrup, capsules, and lozenges, and is employed to alleviate coughs and phlegm [23]. *Rabdosia rubescens* encompasses multiple active constituents, such as diterpenes, triterpenes, steroids, flavonoids, phenolic acids, essential oils, amino acids, alkaloids, and polysaccharides [23]. Research has found that oridonin, a diterpene compound, exhibits significant antitumor activity. It has been found that oridonin can increase the survival rate of esophageal cancer patients [24]. Compounds like ferulic acid, which is present in phenolic acids, and ursolic acid, which is found in triterpenes, possess outstanding anti‐inflammatory properties and are efficacious in treating ailments such as tonsillitis, pharyngitis, and stomatitis [23]. Little research has been conducted into the use of *Rabdosia rubescens* for the treatment of chronic liver disease. Preliminary studies have shown that *Rabdosia rubescens* is also an excellent liver‐protective drug, which has therapeutic effects on nonalcoholic fatty liver disease and other chronic liver diseases by regulating autophagy, lipid metabolism and improving intestinal flora [15, 25, 26]. Based on the excellent effects of *Rabdosia rubescens* in regulating lipid metabolism and autophagy, this paper aims to investigate the therapeutic effects of *Rabdosia rubescens* on ALD.

## 2. Material and Methods

### 2.1. Reagents

*Rabdosia rubescens* was purchased from Guiyang Medicinal Materials Co., Ltd. ALT/GPT test kit, AST/GOT test kit, TCH/T‐CHO test kit, TG test kit, malonaldehyde (MDA) test kit, TBA test kit, interleukin‐1β (IL‐1β) ELISA test kit, and TNF‐α ELISA test kit were purchased from Nanjing Jiancheng Technology Co., Ltd.; BCA protein assay kit and Trizol reagent were purchased from Beijing Leagene Biotechnology Co., Ltd.; and SYBR Primix Ex Taq II kit was purchased from Takara Biotechnology (Dalian) Co., Ltd.

### 2.2. Animals

SPF grade male C57BL/6 mice, aged 6–8 weeks, with Qualification Certificate No.: SCXK (Zhejiang) 2019‐0002, were kept in the animal room of the Laboratory Animal Center at Hangzhou Medical College, under License No.: SYXK (Zhejiang) 2019‐0011. Feeding conditions were maintained as follows: indoor temperature 18°C–26°C, relative humidity 50%–70%, light and dark cycle 12 h/d, and free access to food and water. The mice were housed in cage boxes with corncob bedding. Both the bedding and drinking water were treated with high temperature and high pressure sterilization. Drinking water was supplemented every day. The Lieber–DeCarli standard feed (fat 35%, protein 18%, and carbohydrate 47%) and Lieber–DeCarli alcoholic feed (fat 35%, protein 18%, carbohydrate 19%, and alcohol 28%) were purchased from Trophic Animal Feed High‐tech Co., Ltd. All experiments strictly complied with the People’s Republic of China laws on the use and care of laboratory animals.

### 2.3. Establishment of the Alcoholic Liver Damage Model

A mouse model of alcoholic liver damage was established according to the NIAAA model [27, 28]. The mice were adaptively fed the Liebere–Decarli standard diet for 5 days and then randomly divided into five groups: the control group, the EtOH group, the low dose *Rabdosia rubescens* treatment group (50 mg/kg), the medium dose *Rabdosia rubescens* treatment group (100 mg/kg), and the high dose *Rabdosia rubescens* treatment group (200 mg/kg) with 6 mice in each group. The control group was fed Lieber–DeCarli standard diet, while the other groups were fed Lieber–DeCarli EtOH diet. The treatment groups were also fed the corresponding concentration of Rabdosa rabdosa for 10 days. At 9 am on Day 11, the EtOH group and the treatment groups were given 30% EtOH (5 g/kg) by intragastric administration, while the control group was given an equivalent volume of normal saline. After 9 h, the mice were euthanized, and blood was collected from the abdominal aorta. The blood samples were left at room temperature for 2 h and then centrifuged at 14000 g at 4°C for 10 min. The supernatant was collected and frozen in the refrigerator at −20°C. A portion of the mouse liver was stored in the refrigerator at −80°C, while the remaining part was fixed in formalin.

### 2.4. Detection of Serum Biochemical Indices

Serum samples from the mice were collected and tested using an automatic biochemical analyzer according to the instructions of ALT, AST, TG, TC, and TBA test kits.

### 2.5. Detection of MDA, TBA, and TC Contents in Mouse Liver Tissues

Liver tissues stored at −80°C were removed from the refrigerator and thawed on ice. Residual blood was removed using precooled PBS. A 100‐mg sample of liver tissue was extracted using a chloroform–methanol mixture (2:1 vol/vol) to extract the liver bile acids, which were then detected according to the instructions of the TBA test kit. The liver tissues were homogenized. According to the instructions of the MDA and TC test kits, the absorption peak at the specified wavelength was detected using an ultraviolet spectrophotometer. The contents of MDA, TBA, and TC in the liver tissue homogenate of mice in each group were calculated using the calculation formula in the kit instructions.

### 2.6. Immunohistochemistry

Decalcify the sections using a decalcification solution and then rehydrate them by immersing them in alcohol of different concentrations. Heat to 100°C for 20 min to achieve antigen retrieval. Incubate the sections in hydrogen peroxide for 5 minutes to reduce endogenous peroxidase activity. Add the LC3B and p62 antibodies and incubate the sections at room temperature for 30 min. Next, incubate the sections in a polymer‐conjugated horseradish peroxidase IgG reagent for 8 minutes to detect the primary antibody. Use diaminobenzidine tetrahydrochloride as the substrate to detect the antigen–antibody reaction. Finally, stain with hematoxylin for 5 minutes to recolor the cell nuclei. The level of LC3B and p62 staining was determined by the histoscore (H‐score) calculated by the multiplication of intensity score (0 = none, 1 = weak, 2 = moderate, and 3 = strong) with values representing the percentage of positively stained cells (0 ≤ 10%; 1 = 10–25%; 2 = 25–50%; 3 = 50–75%; and 4 ≥ 75%) [29].

### 2.7. Hematoxylin and Eosin (H&E) Stain

The formalin‐fixed mouse liver tissues were dehydrated in alcoholic solution of varying concentrations and cleared in xylene solution. The tissues were then embedded in melted paraffin wax, cooled, and sliced using a microtome. The sections were mounted on slides, stained with H&E, dehydrated with alcohol, cleared in xylene, and sealed.

### 2.8. Expression of Liver Inflammatory Factors Detected With ELISA

The liver homogenate was diluted following the instructions of the ELISA test kit. The standard product and sample were then added to the plate and incubated at 37°C for 30 min. After washing, an enzyme‐labeled reagent was added and incubated at 37°C for 30 min. A chromogenic reagent was added to develop color for 15 min. Then, a termination solution was added to stop the reaction. The absorption peak at 450 nm was detected immediately using a microplate reader. The expression levels of TNF‐α and IL‐1β was calculated according to the instructions.

### 2.9. Expression of Fat Metabolizing Enzymes and Autophagy‐Related Proteins Detected Using the Western Blot Method

The protein concentration of mouse liver homogenate was determined using the BCA method. The sample buffer was prepared and added into the electrophoresis tank for gel electrophoresis. The gel was then transferred to a membrane in a sandwich structure for transmodeling. The membrane was blocked with skim milk powder, followed by incubation with primary and secondary antibodies. Subsequently, a luminescence reaction was performed in a dark room and exposed using a fully automatic gel imaging system. CYP7A1, SREBP, FAS, LC‐3, Beclin‐1, p62, and ATG7 protein band images were saved, and the gray value of the protein bands was analyzed using ImageJ Software. The antibodies are shown in Table 1.

**Table 1:** Primary antibodies in Western blot.

Name	Item number	Molecular weight (kDa)	Dilution	Host	Manufacturer
CYP7A1	18054‐1‐AP	58	1/3000	Rabbit	Proteintech
SREBP	14088‐1‐AP	125	1/2000	Rabbit	Proteintech
FAS	10624‐2‐AP	272	1/5000	Rabbit	Proteintech
LC‐3B	14600‐1‐AP	15	1/5000	Rabbit	Proteintech
Beclin‐1	11306‐1‐AP	52	1/5000	Rabbit	Proteintech
ATG7	10088‐2‐AP	78	1/1000	Rabbit	Proteintech
P62	18420‐1‐AP	48	1/10,000	Rabbit	Proteintech
GAPDH	10494‐1‐AP	36	1/10,000	Rabbit	Proteintech

### 2.10. Expression of Fat Metabolizing Enzymes and Autophagy‐Related Proteins Detected Using the RT‐qPCR Method

The RNA of the liver homogenate was extracted using Trizol reagent, followed by extraction with trichloromethane, centrifugation, and precipitation with isopropyl alcohol. Ethanol was then added for air drying. The purity and concentration of the RNA were determined, and the RNA was reverse‐transcribed to obtain cDNA. According to SYBR Primix Ex Taq II kit, the cDNA template was amplified by RT‐PCR with gene‐specific primers. The data were quantitatively analyzed using the 2^−△△CT^ method. The primer sequence is shown in Table 2.

**Table 2:** Primer sequence.

Gene	Sequence
CYP7A1	5′‐AAC​AAC​CTG​CCA​GTA​CTA​GAT​AGC‐3′
	5′‐GTG​TAG​AGT​GAA​GTC​CTC​CTT​AGC‐3′

SREBP	5′‐TGC​ATT​TTC​TGA​CAC​GCT​TC‐3′
	5′‐CCA​AGC​TGT​ACA​GGC​TCT​CC‐3′

FAS	5′‐CAC​CCG​GAC​CCA​GAA​TAC​C‐3′
	5′‐TGT​TGC​TGG​TGA​GTG​TGC​ATT‐3′

LC‐3	5′‐GGC​GTC​TTT​GTG​GGT​TGG‐3′
	5′‐GCC​TGC​TTG​TCC​TGG​TTG‐3′

Beclin‐1	5′‐GGA​TGG​ATG​TGG​AGA​AAG​GCA​AG‐3′
	5′‐TGA​GGA​CAC​CCA​AGC​AAG​ACC‐3′

ATG7	5′‐TCG​AAA​GCC​ATG​ATG​TCG​TCT​T‐3′
	5′‐CCA​AAG​CAG​CAT​TGA​TGA​CCA‐3′

P62	5′‐CTG​GGA​CTG​AGA​AGG​CTC​AC−3′
	5′‐GCA​GCT​GAT​GGT​TTG​GAA​AT‐3′

GAPDH	5′‐GAA​GGT​GAA​GGT​CGG​AGT​C‐3′
	5′‐GAA​GAT​GGT​GAT​GGG​ATT​TC‐3′

### 2.11. Statistical Analysis

SPSS 23.0 Software was used for statistical analysis, and a histogram was drawn using GraphPad Software. Protein bands were processed using Image J. Measurement data were expressed as $\overset{¯}{x}$ ± *s*, and pairwise comparison was performed using the LSD‐*t* test, with a test level of *α* = 0.05.

## 3. Results

### 3.1. *Rabdosia Rubescens* Improves Lipid Metabolism Disorders and Inhibits Hepatocyte Apoptosis in ALD Mice

Serum biochemical indices, liver MDA levels, and H&E‐stained liver section results of mice are shown in Figure 1. Following the administration of the Lieber–DeCarli alcoholic feed and a single high‐dose of EtOH treatment to mice, the serum ALT and AST levels, as well as liver MDA levels of mice, significantly increased, showing statistical significance (*p* < 0.05). H&E staining results revealed extensive apoptosis around the central vein of the liver. Relevant indices of fat metabolism are shown in Figure 2. After establishing the ALD model, the TG and TC contents in the serum of mice significantly increased, along with a notable increase in liver TC content, indicating statistical significance (*p* < 0.05). Biochemical and lipid metabolism indices confirm the successful establishment of the ALD model. The levels of ALT, AST, TG, and TC in serum significantly decreased in the ALD mouse model treated with *Rabdosia rubescens* for 10 days (*p* < 0.05); the levels of MDA and TC in the liver significantly decreased (*p* < 0.05), and the apoptosis of hepatocytes significantly improved in H&E‐stained sections. The results showcase that *Rabdosia rubescens* has protective effects on hepatocytes of ALD mice and reduces fat production in these mice. The test results of lipid metabolism‐related enzymes, assessed using Western Blot and qPCR, are shown in Figure 2. The SREBP and FAS protein and mRNA levels significantly decreased after administration of *Rabdosia rubescens*, showing statistical significance (*p* < 0.05), suggesting that *Rabdosia rubescens* can reduce liver fat synthesis and alleviate liver steatosis in ALD mice and has protective effects on ALD.

Figure 1Effects of *Rabdosia rubescens* on liver damage in ALD mice. Note: (a–c) the results of serum biochemical indices. (a) Serum ALT; (b) serum AST; and (c) liver MDA value; ^∗^: *p* < 0.05 compared with the model group. (d–h) The results of H&E staining. (d) Control group; (e) EtOH group; (f) *Rabdosia rubescens* 200 mg/kg group; (g) *Rabdosia rubescens* 100 mg/kg group; and (h) *Rabdosia rubescens* 50 mg/kg group, observed at 200x magnification.

**Figure:**
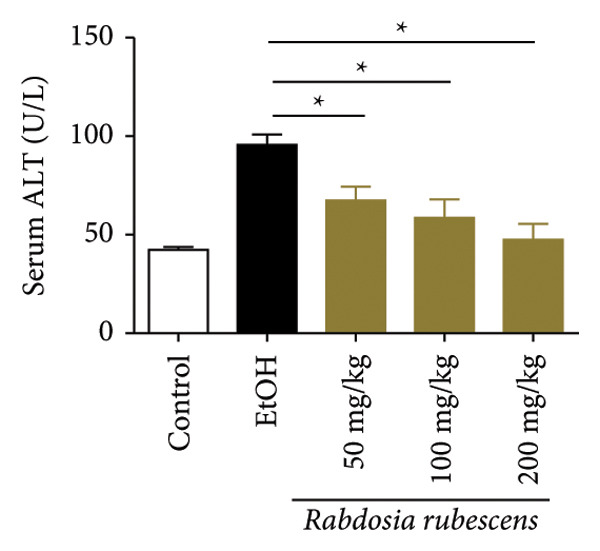
(a)

**Figure:**
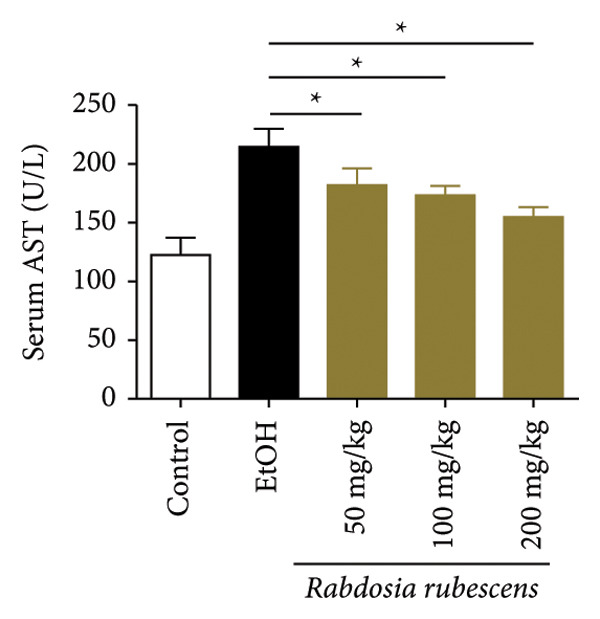
(b)

**Figure:**
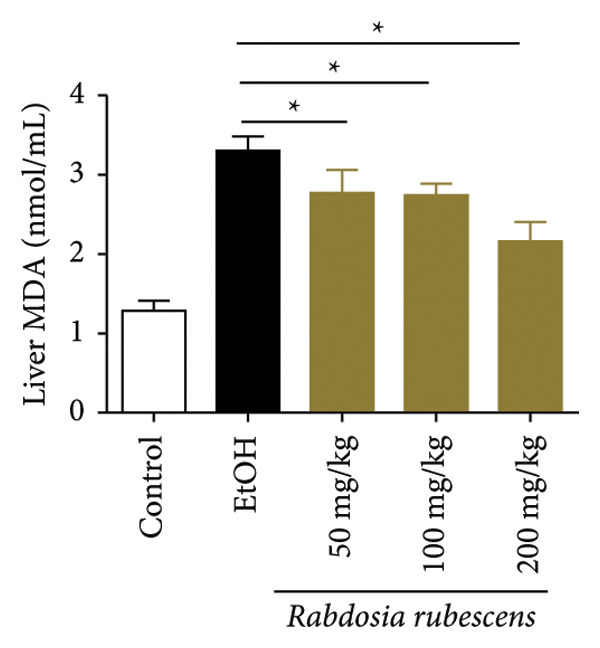
(c)

**Figure:**
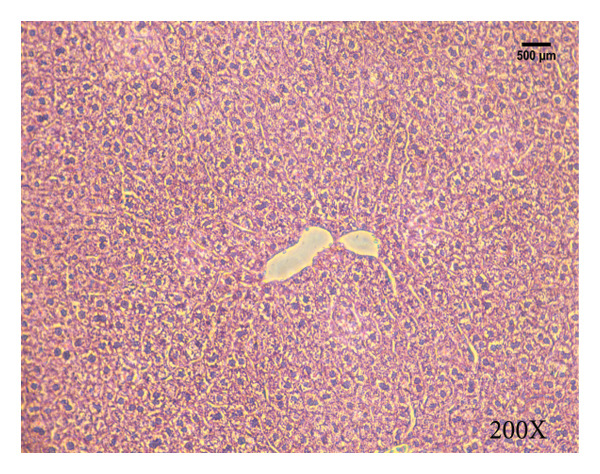
(d)

**Figure:**
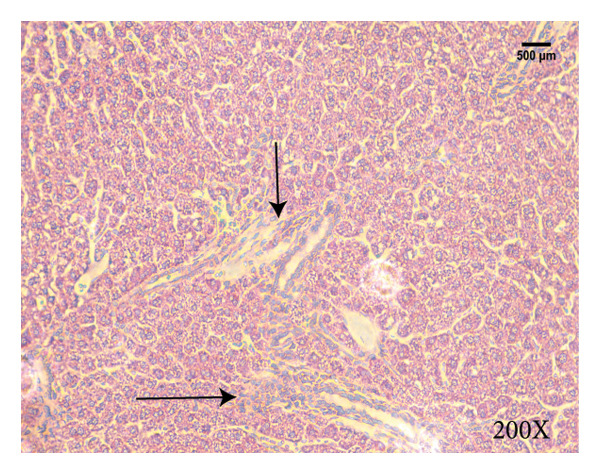
(e)

**Figure:**
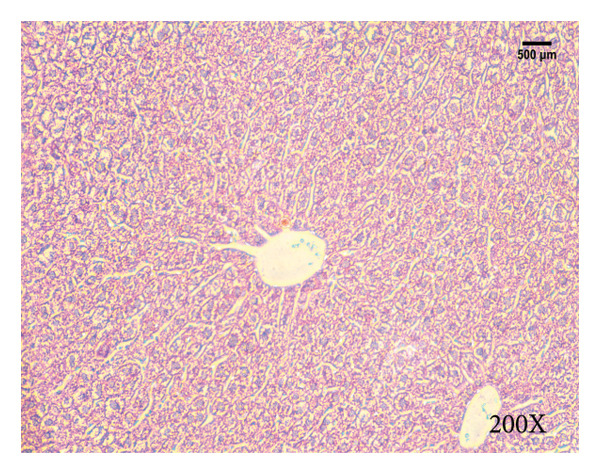
(f)

**Figure:**
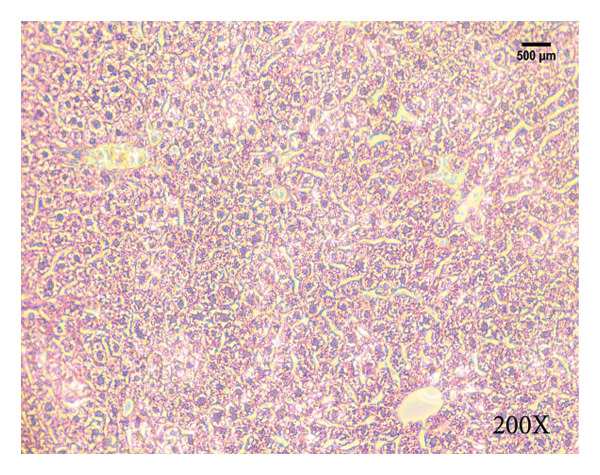
(g)

**Figure:**
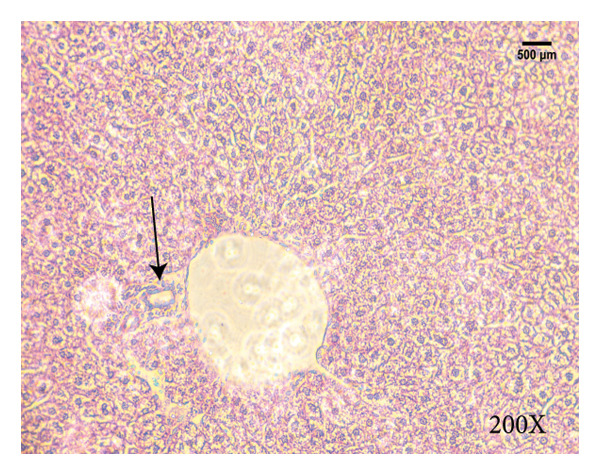
(h)

Figure 2Effects of liver fat metabolism related indices in ALD mice. Note: (a–c) the test results of biochemical indices. (a) Serum TG; (b) serum TC; and (c) liver TC. (d–h) The results of Western blot and qPCR. (d) SREBP and FAS protein bands; (e‐f) quantified gray values of SREBP and FAS protein bands using ImageJ software; and (g‐h) mRNA expression of SREBP and FAS; ^∗^: *p* < 0.05 compared with the model group.

**Figure:**
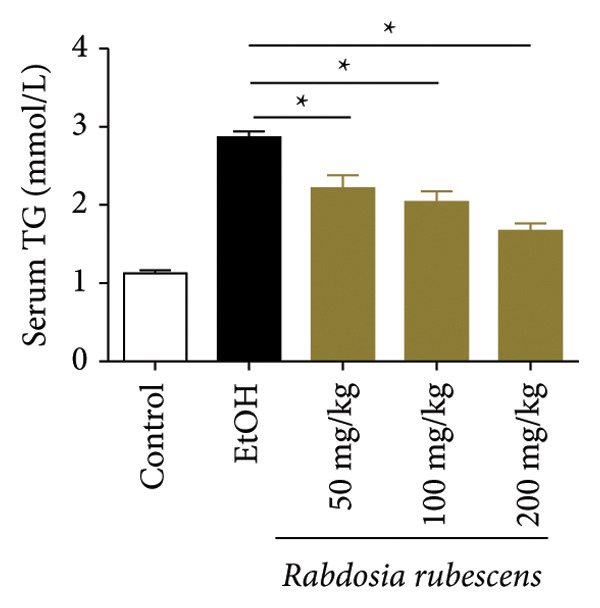
(a)

**Figure:**
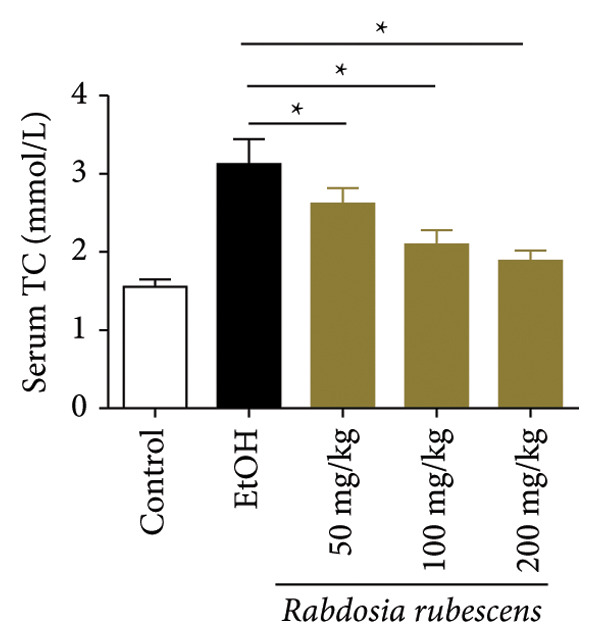
(b)

**Figure:**
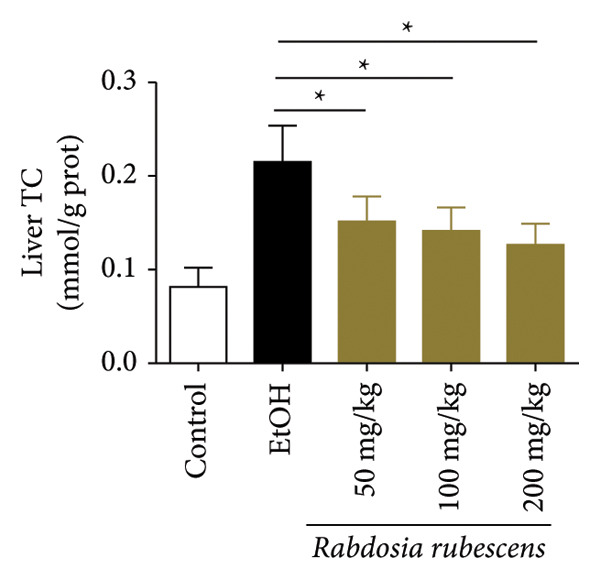
(c)

**Figure:**
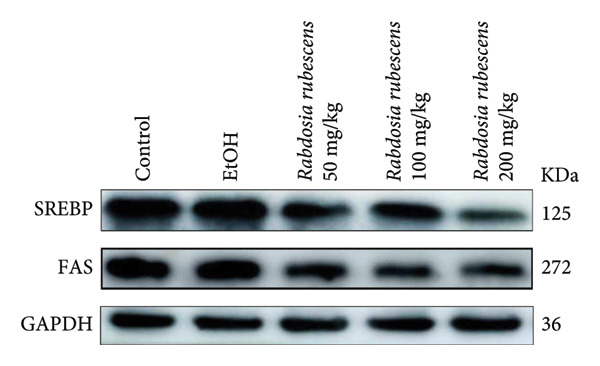
(d)

**Figure:**
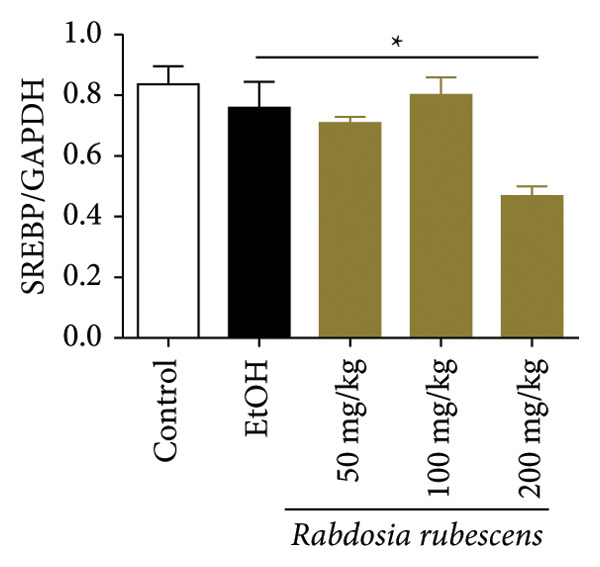
(e)

**Figure:**
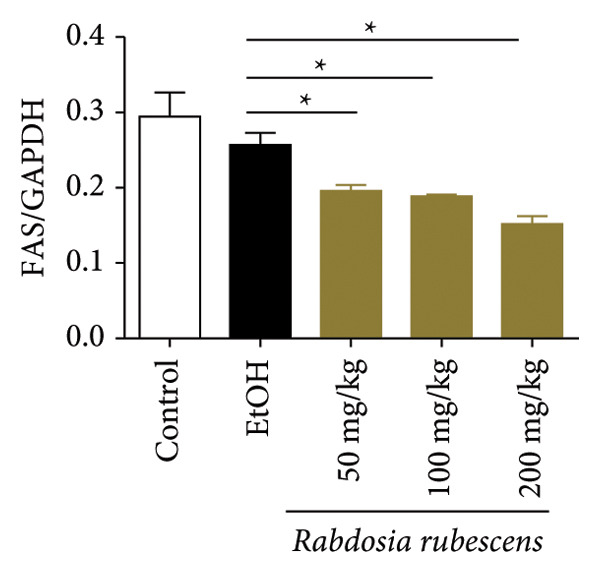
(f)

**Figure:**
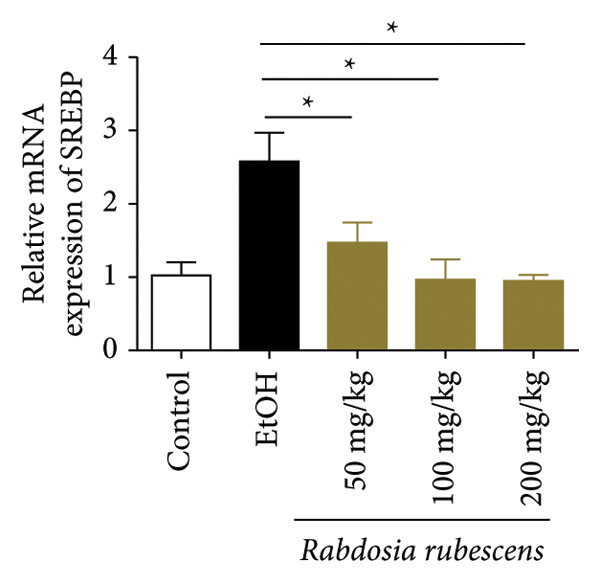
(g)

**Figure:**
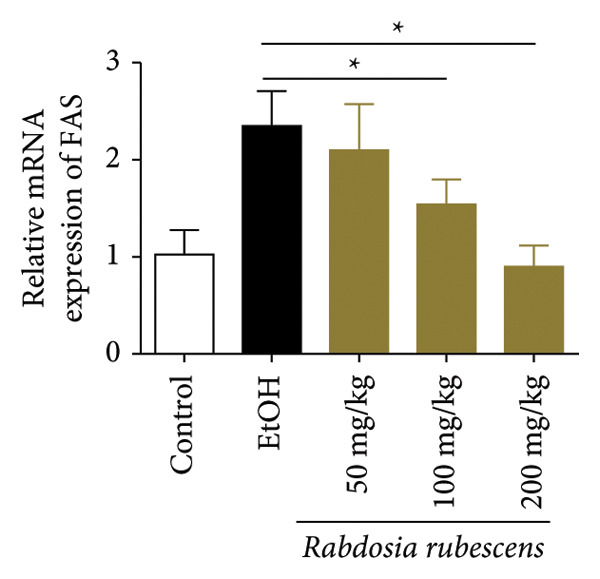
(h)

### 3.2. *Rabdosia Rubescens* Improves Liver Cholestasis in ALD Mice by Inhibiting CYP7A1 Expression

The test results of serum and liver TBA contents in ALD mice are as shown in Figure 3. The serum and liver TBA levels in the model group mice were significantly increased but showed a notable decrease after treatment with *Rabdosia rubescens*, showing statistical significance (*p* < 0.05). As shown in Figure 3, the expression of liver inflammatory factors TNF‐α and IL‐1β was significantly reduced after treatment with *Rabdosia rubescens* (*p* < 0.05). These results align with the change in TBA content, indicating that cholestasis was alleviated and liver inflammation caused by cholestasis was mitigated. Furthermore, the expression of CYP7A1 was analyzed using Western Blot and qPCR methods. As shown in Figure 3, CYP7A1 expression was significantly inhibited after treatment with *Rabdosia rubescens*. It indicates that *Rabdosia rubescens* can suppress CYP7A1 expression, reduce bile acid production, relieve liver cholestasis, and alleviate liver inflammation.

Figure 3Effects of *Rabdosia rubescens* on bile acid metabolism in ALD mice. Note: (a‐b) bile acid content results. (a) Serum bile acid content and (b) liver bile acid content. (c–e) The results of Western blot and qPCR. (c) CYP7A1 protein band; (d) quantified gray value of CYP7A1 protein band with ImageJ software; and (e) mRNA expression of CYP7A1. (f‐g) ELISA test results. (f) Liver IL‐1β and (g) liver TNF‐α; ^∗^: *p* < 0.05 compared with the model group.

**Figure:**
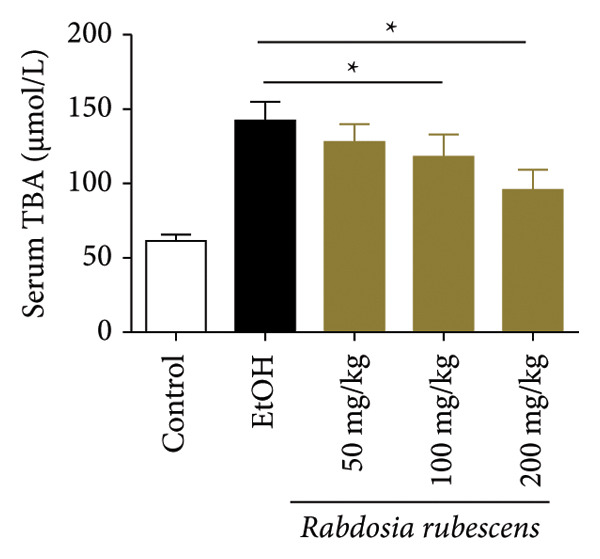
(a)

**Figure:**
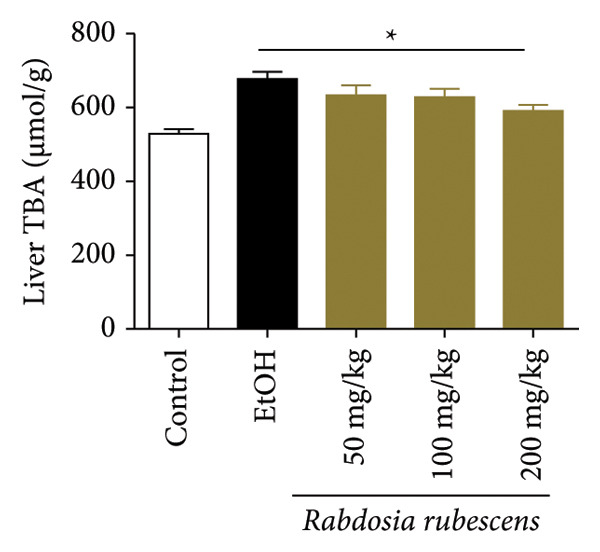
(b)

**Figure:**
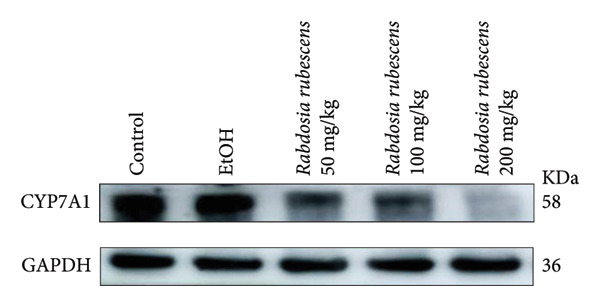
(c)

**Figure:**
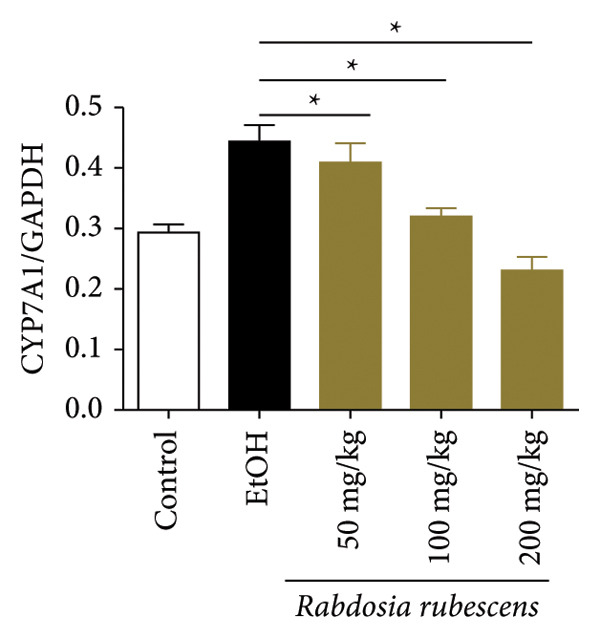
(d)

**Figure:**
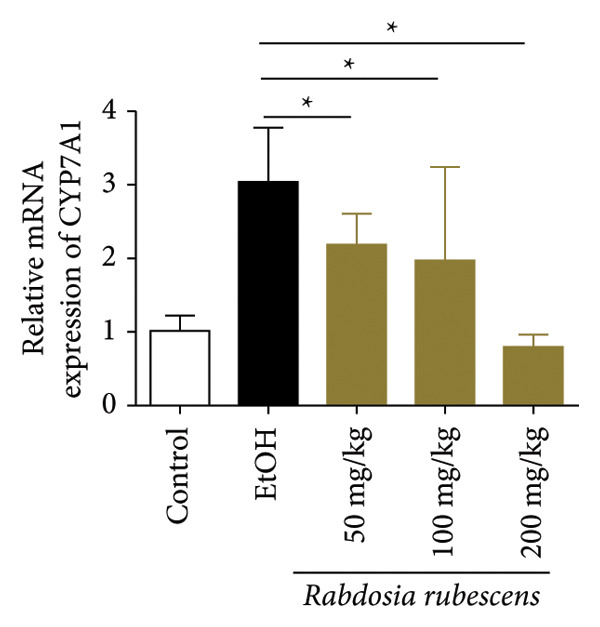
(e)

**Figure:**
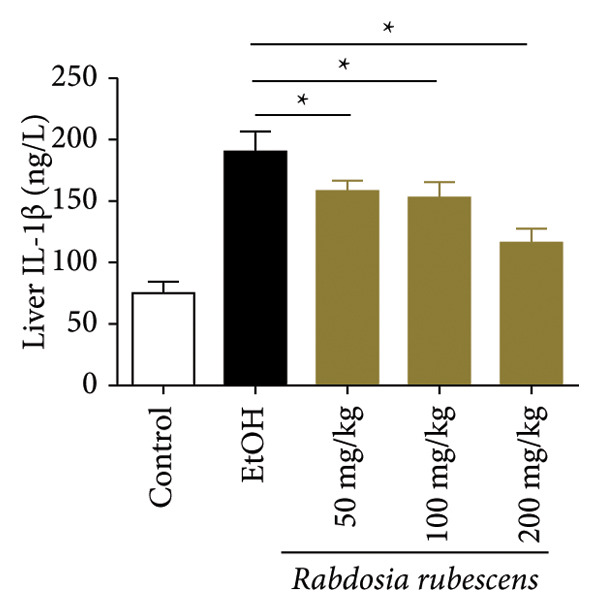
(f)

**Figure:**
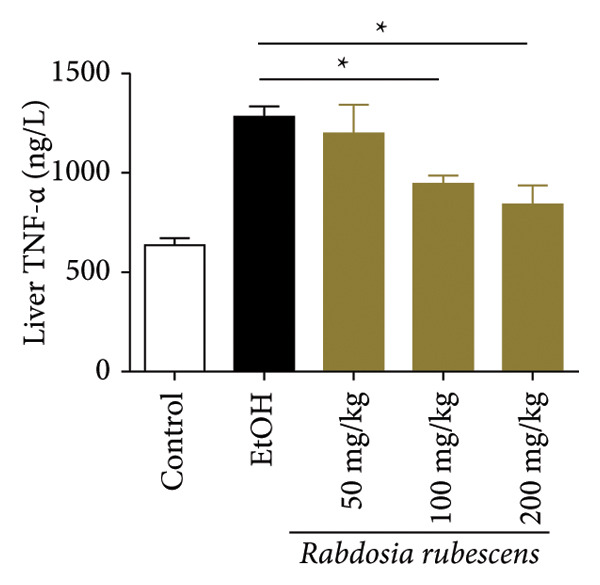
(g)

### 3.3. *Rabdosia Rubescens* Improves Liver Damage in ALD Mice by Inducing Autophagy

Immunohistochemical staining was used to detect the expression of LC3 and p62 in mice livers. The results are shown in Figure 4. LC‐3 staining intensity was low positive in the model group, while p62 staining intensity was positive. This indicates that the level of autophagy in the mouse liver increased slightly under alcohol induction. Compared with the model group, mice treated with *Rabdosia rubescens* showed high positive LC3 staining and LC3 puncta in cytoplasmic, while p62 staining was low positive. These results suggest that wintergreen can activate autophagy. The Western Blot and qPCR were used to test the expression of autophagy related proteins, as shown in Figure 5. In the model group, the expression levels of LC‐3, Beclin‐1, and ATG7 increased, whereas the expression level of p62 decreased, suggesting that the ALD body can promote hepatocyte survival by upregulating the autophagy level, facilitating the phagocytosis of damaged mitochondria and excessive lipid droplets in hepatocytes. The protein and mRNA levels of LC‐3, Beclin‐1, and ATG7 significantly increased after treatment with *Rabdosia rubescens* (*p* < 0.05), whereas the expression level of p62 decreased, suggesting that *Rabdosia rubescens* can upregulate the autophagy level and enhance the self‐protective ability of hepatocytes.

Figure 4The results of LC3B and p62 immunohistochemical stains in ALD mice. Note: (a) the results of LC3B and p62 immunohistochemical. (b) The LC3 and P62 H‐score experimental results. ^∗^: *p* < 0.05 compared with the model group, observed at 400x magnification.

**Figure:**
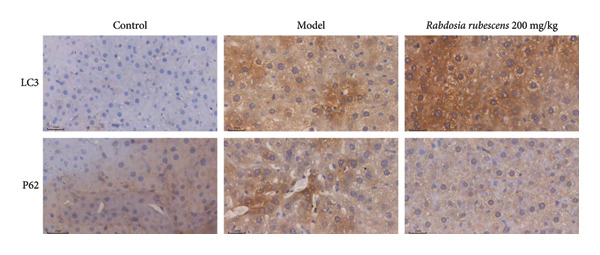
(a)

**Figure:**
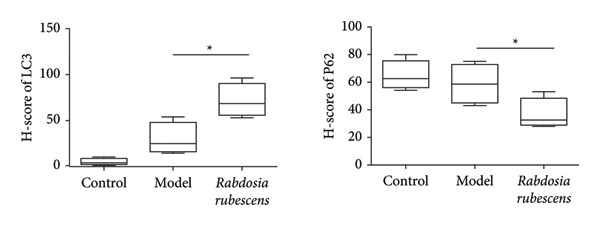
(b)

Figure 5Effects of *Rabdosia rubescens* on autophagy in ALD mice. Note: (a) LC‐3, ATG7, P62, and Beclin‐1 protein bands and (b–e) quantified gray values of protein bands with ImageJ software. (b) LC‐3; (c) ATG7; (d) Beclin‐1; and (e) P62. (f–i) The test results of qPCR. (f) LC‐3; (g) ATG7; (h) Beclin‐1; and (i) P62; ^∗^: *p* < 0.05 compared with the model group.

**Figure:**
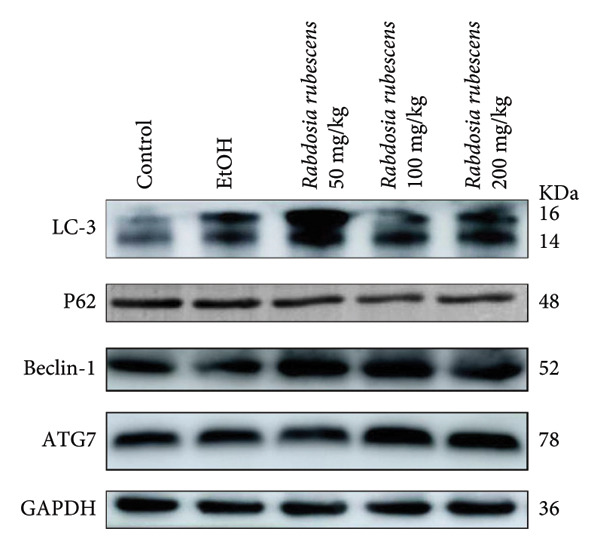
(a)

**Figure:**
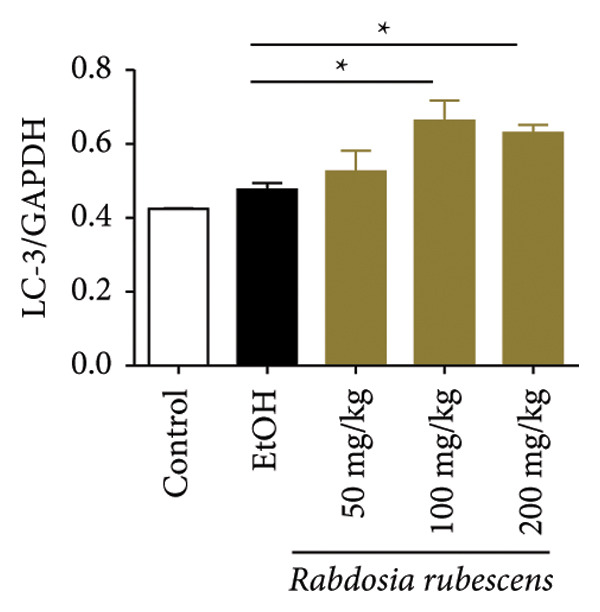
(b)

**Figure:**
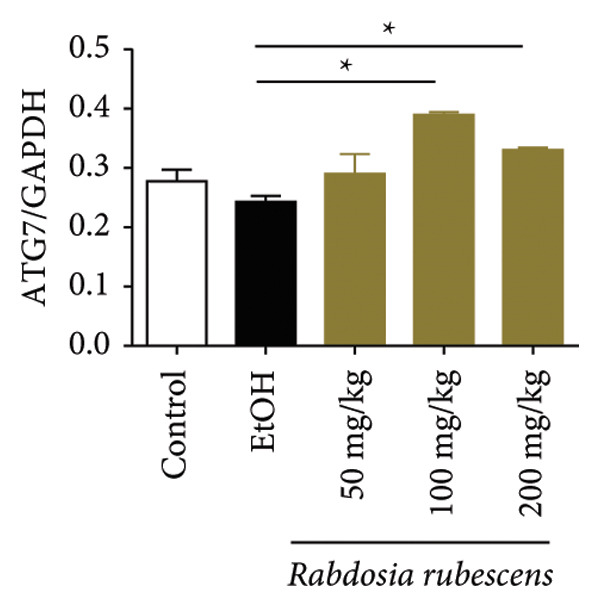
(c)

**Figure:**
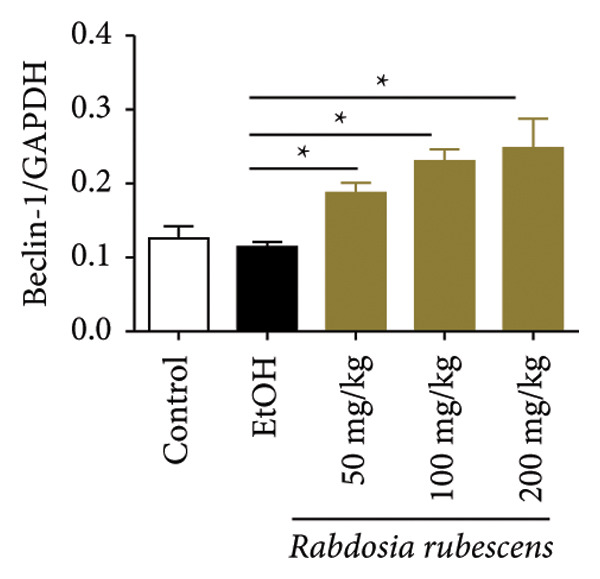
(d)

**Figure:**
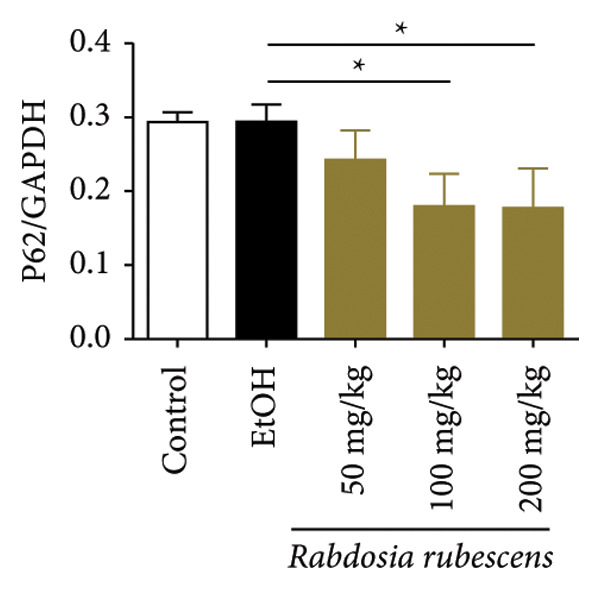
(e)

**Figure:**
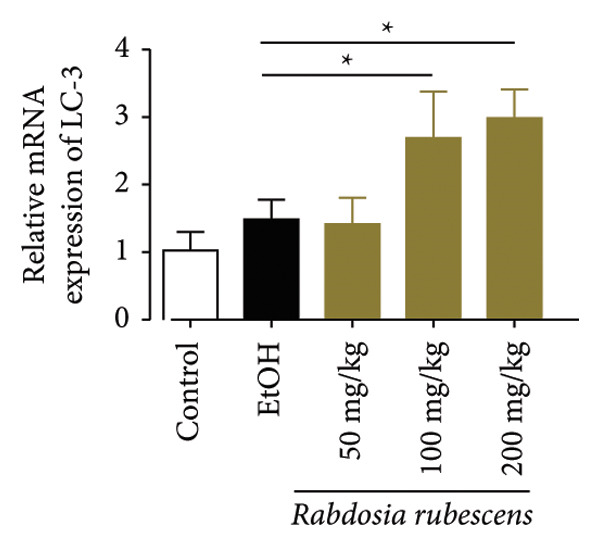
(f)

**Figure:**
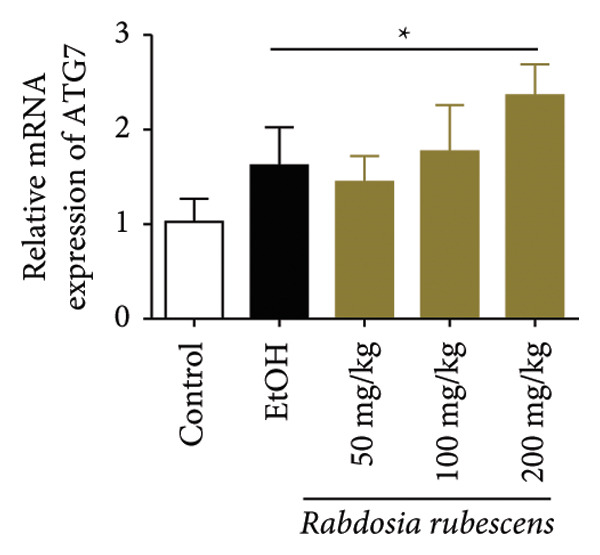
(g)

**Figure:**
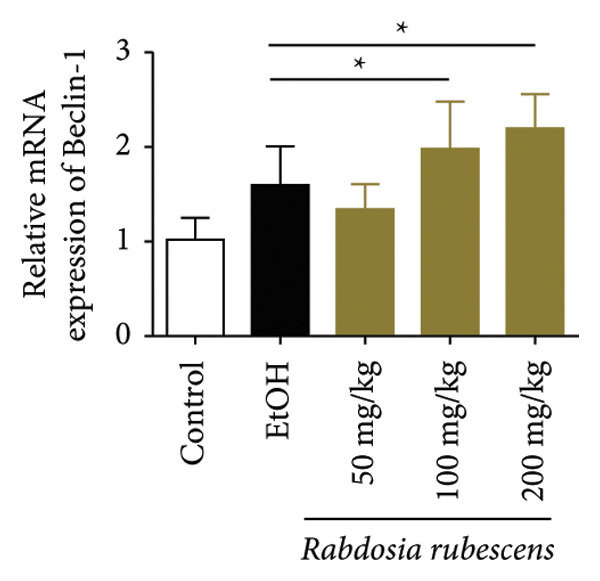
(h)

**Figure:**
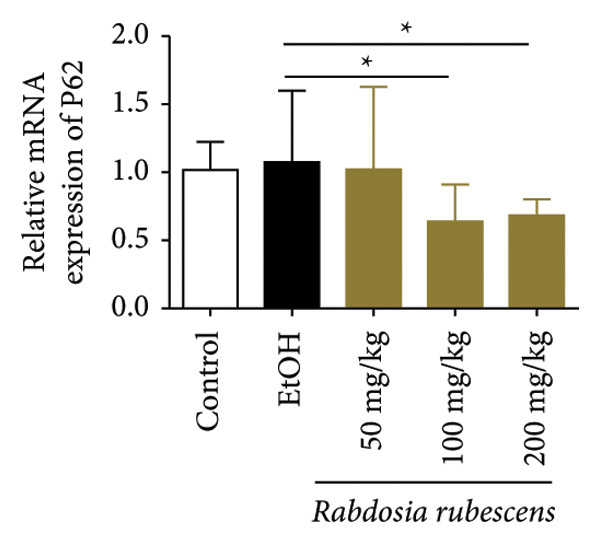
(i)

## 4. Discussion

The effects of long‐term alcohol consumption on the liver manifest in multiple ways [9, 17]. First of all, it significantly impacts lipid metabolism. Excessive NADH produced by ethanol during metabolism inhibits the oxidation of fatty acids, reduces fatty acid output, and promotes fat accumulation [2]. The accumulation of fatty acids provides raw materials for the synthesis of CHO and bile acids, while the overexpression of CYP7A1 provides the driving force for the synthesis of bile acids [20], which is a key cause of cholestasis in ALD patients. FXR inhibits the synthesis of bile acids by recognizing excessive bile acids [30]. However, alcohol consumption lowers NAD + levels and inactivates NAD^+^‐dependent sirtuins1 (SIRT1) [31]. This leads to increased FXR acetylation and impaired activity. The expression level of bile acid CoA: amino acid N‐acyltransferase is significantly reduced in ALD patients, leading to incomplete bile acid metabolism and the production of more toxic intermediates [17]. Second, ALD patients often exhibit evident hepatitis [32], which may result from reduced secretion of intestinal oxidative stress mediated antimicrobial peptides and the destruction of the intestinal barrier caused by alcohol consumption [33]. This disruption allows pathogens to invade the liver. Excess bile acids and intermediates can also directly induce inflammation [17]. Finally, during the development of ALD, the liver can activate autophagy to phagocytize excess fat droplets and damaged mitochondria in liver cells, promoting its own survival [34]. Therefore, regulating fat metabolism, bile acid synthesis, and autophagy could serve as potential targets for ALD treatment. Preliminary studies have shown that *Rabdosia rubescens* can regulate lipid metabolism and induce autophagy, but these effects have not yet been explored in ALD. Therefore, this paper aims to investigate the therapeutic effects and mechanisms of *Rabdosia rubescens* in ALD.

We first established a mouse model of ALD in a chronic‐plus‐binge drinking manner [28, 35]. Mice were fed Lieber–DeCarli alcoholic diet for 10 days, followed by a high‐concentration ethanol dose. This method effectively simulated the metabolic disorders and liver cell apoptosis in long‐term alcohol consumption patients. Mice in the treatment groups were fed the Lieber–DeCarli alcoholic diet and treated with *Rabdosia rubescens*. The results showed that *Rabdosia rubescens* significantly reduced the levels of ALT, AST, and MDA in the serum of mice. Additionally, the number of apoptotic cells in H&E‐stained sections significantly decreased, suggesting that *Rabdosia rubescens* inhibits hepatocyte apoptosis in ALD mice. We then tested fat metabolism, and the results indicated that *Rabdosia rubescens* reduced serum TG, TC, and liver TC levels in ALD mice. We further tested the expression of proteins related to fatty acid synthesis in the liver of mice, and the results indicated that *Rabdosia rubescens* reduced the expression of SREBP and FAS. Therefore, *Rabdosia rubescens* can inhibit fat synthesis and improve alcohol‐induced liver damage. Following this, we tested bile acid content and liver inflammation in ALD mice. The results demonstrated that treatment with *Rabdosia rubescens* significantly decreased serum and liver TBA levels, reduced the expression of liver inflammatory factors TNF‐α and IL‐1β, and alleviated cholestasis. This improvement may also be attributed to the inhibition of fat metabolism by *Rabdosia rubescens*. Therefore, we also tested the expression of CYP7A1, a key enzyme in bile acid synthesis, and found that *Rabdosia rubescens* inhibited CYP7A1 expression, thereby reducing the synthesis of bile acids. Finally, we tested the effect of *Rabdosia rubescens* on the liver autophagy level in ALD mice. The liver autophagy level in ALD mice itself increased to a certain extent, but this increase became more evident after treatment with *Rabdosia rubescens*. The results indicate that the expression of LC3, Beclin‐1, and ATG7 significantly increased after treatment with *Rabdosia rubescens*, leading to a notable enhancement of autophagy. Therefore, we can preliminarily conclude that *Rabdosia rubescens* can treat ALD by reducing lipid synthesis, particularly bile acid synthesis, and upregulating autophagy.

## Disclosure

All authors have read and approved the final manuscript.

## Conflicts of Interest

The authors declare no conflicts of interest.

## Author Contributions

Xi Zou performed lab work and wrote the original draft. Tingting Shi and Yunling Ke conceptualized the study and proposed the methodology. Wei Chen and Yidan Shao conducted Western Blot and RT‐qPCR experiments.

## Funding

This study was supported by the Hangzhou Joint Fund of the Zhejiang Provincial​ Natural Science Foundation of China under Grant no. LHZY24H310001; the Science and Technology Bureau of Hangzhou Grant nos. 20220919Y039, 20220919Y042, and 20241029Y048; and Hangzhou Medical and Health Science and Technology Project (no: A20240834).

## Data Availability

The data that support the findings of this study are available from the corresponding author upon reasonable request.
